# FAIR, safe and high-quality data: The data infrastructure and accessibility of the YOUth cohort study

**DOI:** 10.1016/j.dcn.2020.100834

**Published:** 2020-08-05

**Authors:** Jelmer J. Zondergeld, Ron H.H. Scholten, Barbara M.I. Vreede, Roy S. Hessels, A.G. Pijl, Jacobine E. Buizer-Voskamp, Menno Rasch, Otto A. Lange, Coosje L.S. Veldkamp

**Affiliations:** a Experimental Psychology, Helmholtz Institute, Utrecht University, the Netherlands; b Utrecht University Library, Utrecht University, the Netherlands; c Developmental Psychology, Utrecht University, the Netherlands; d University Medical Center Utrecht, the Netherlands; e Faculty of Social Sciences, Utrecht University, the Netherlands; f Information and Technology Services, Utrecht University, the Netherlands

**Keywords:** UU, Utrecht University, CRC, Child Research Center, UMCU, University Medical Center Utrecht, ITS, Utrecht University Information and Technology Services, SLIM, Study Logistics and Information Manager, RO, Research Online, RDP, Research Data Platform, WUR, Wageningen University, RIA, Research Imaging Architecture, GDPR, General Data Protection Regulation, Information technology, Research data management, Data infrastructure, Open science, FAIR data, Cohort study

## Abstract

The YOUth cohort study aims to be a trailblazer for open science. Being a large-scale, longitudinal cohort following children in their development from gestation until early adulthood, YOUth collects a vast amount of data through a variety of research techniques. Data are collected through multiple platforms, including facilities managed by Utrecht University and the University Medical Center Utrecht. In order to facilitate appropriate use of its data by research organizations and researchers, YOUth aims to produce high-quality, FAIR data while safeguarding the privacy of participants. This requires an extensive data infrastructure, set up by collaborative efforts of researchers, data managers, IT departments, and the Utrecht University Library. In the spirit of open science, YOUth will share its experience and expertise in setting up a high-quality research data infrastructure for sensitive cohort data. This paper describes the technical aspects of our data and data infrastructure, and the steps taken throughout the study to produce and safely store FAIR and high-quality data. Finally, we will reflect on the organizational aspects that are conducive to the success of setting up such an enterprise, and we consider the financial challenges posed by individual studies investing in sustainable science.

## Highlights

• YOUth is a longitudinal cohort study in the Netherlands that aims to produce and safely store FAIR and high-quality data.
• We share our experience and expertise in setting up a high-quality research data infrastructure for sensitive cohort data.
• We describe our procedures and the technical aspects of our data and data infrastructure.
• We highlight the importance of collaboration between organizations.

## 1 Introduction

### 1.1 The YOUth study

YOUth (Youth of Utrecht) is a large-scale, longitudinal cohort study following children (and their parents) from the area of Utrecht, the Netherlands, from gestation to adolescence. The rationale and design of the YOUth study are provided by Onland-Moret et al. (in press, this issue) test and are briefly summarized in this section.

The primary aim of the YOUth cohort study is to understand the development of social competence—the “ability to engage in meaningful interaction with others”—and self-regulation—“the ability to control one’s emotions, behaviour and impulses and to adapt to rules”. These competencies are thought to rely on various neurocognitive abilities. The interaction of biological, child-related and environmental factors guides the development of these neurocognitive abilities. This interaction, however, is poorly understood. The YOUth cohort gathers data that may enable the construction of predictive models of behaviour from environmental and biological determinants by measuring biological, child-related and environmental factors, as well as abilities and competencies throughout development and in various contexts.

The cohort is divided into two sub-cohorts, focusing on different age groups: YOUth Baby & Child, from gestation to around the age of six, and YOUth Child & Adolescent, from around the age of nine to around the age of 15. The aim is to include 3000 and around 2000 children and their parents in these two sub-cohorts respectively. Inclusion for the sub-cohorts is done in parallel. Participants visit our Child Research Center (CRC) at regular intervals (“waves”) and measurements are repeated at each wave. Participants are recruited in various ways, e.g. through hospitals, midwifery practices, daycare centres, and primary schools. Recruitment began in July 2015 and is ongoing.

YOUth collects biological material and body measurements, and measurements of children's development through computer tasks, questionnaires, eye-tracking experiments, EEG experiments, (f)MRI scans, video recordings of parent-child interaction, 3D ultrasound sweeps of the foetal brain and video tasks (see for a full description test Onland-Moret et al., in press, this issue).

### 1.2 YOUth’s ambitions regarding research data

YOUth aims to be a trailblazer for open science. Part of this ambition constitutes the production and safekeeping of FAIR and high-quality data. Here, we describe the objectives for data FAIRness, safety and quality, look ahead at the importance of collaborative effort, and outline the structure of this paper.

The FAIR principles state that data should be findable, accessible, interoperable and reusable (Wilkinson et al., 2016). They enable the scientific community at large to extract maximum benefit from the research data that we generate, and thereby to treat publicly funded time and effort responsibly (Mons, 2018). The basis of our approach, and an integral part of the implementation of the FAIR principles, is proper data management and a suitable infrastructure. In addition, we invest in rich metadata, facilitating both the potential findability and reusability of our data, and we use interoperable formats where possible. These principles guide our navigation between the safekeeping of highly privacy-sensitive data on the one hand and facilitating the scientific community’s access to these rich and unique data on the other hand. We elaborate on our approach to FAIR data in Section 2.

High safety standards are another important aim of YOUth. Our research data include information from children and their parents, which are by nature privacy-sensitive and must be shared with caution and bear adequate protection against unauthorized access. Moreover, the majority of the data collected is personally identifiable, either indirectly by being linkable to a particular person, or directly (e.g. video recordings or certain questionnaire responses). Some, such as genetic data and information on religious beliefs, are legally classified as special categories of personal data by European legislation (General Data Protection Regulation (GDPR) art. 9). These data should thus be processed with particular care, requiring extensive technical and legal measures to ensure the privacy of participants.

Finally, we aim to provide high-quality data. We investigate children from a wide age range using a plethora of research methods and procedures that are often technically complex and may be prone to error or malfunction. YOUth therefore employs extensive procedures regarding data collection and monitoring of data quality to ensure that high-quality data are collected.

Realizing these three ambitions requires collaborative efforts of multiple parties. The YOUth cohort is a joint research effort by a number of research organizations. Therefore, the data infrastructure in which these procedures are realized must be a joint effort as well, combining components of different organizations into a unified and integrated system. Furthermore, partner organizations with the necessary expertise, personnel and resources to facilitate research data at scale had to be included. In our case, we partnered with (among others) the Utrecht University Library (UUL) and the Utrecht University Information and Technology Services department (ITS).

In the spirit of open science, YOUth will share its experience and expertise in collaboratively setting up a high-quality research data infrastructure for sensitive cohort data. In this paper, we present a model of data quality control and data stewardship that meets the criteria outlined above and puts YOUth, as a large and complex cohort study, at the forefront of proper data infrastructure and management procedures, and at the forefront of using the FAIR principles in the context of sensitive cohort data. In what follows, we will explain how the development of our data infrastructure was guided by the FAIR principles as well as data safety and quality goals. First, we will discuss how we applied the FAIR principles, as this required the largest infrastructural investments. Next, we will describe additional measures we took to ensure optimal safety and confidentiality. Finally, we will discuss the procedures we developed to enhance data quality and close with a discussion on the lessons we learned throughout this endeavour as well as future opportunities and challenges.

## 2 FAIR data

In recent years, academic institutions and funding agencies, such as the Dutch Research Council (NWO, n.d.), have been making their requirements regarding data FAIRness, archiving, and publication stricter, and have developed tools through which these requirements can be met. The Utrecht University Open Science Programme (Utrecht University Open Science Task Force, 2018) specifically targets the findability of research data, and Utrecht University facilitates FAIR storage and archiving of research data through the Yoda research data management system (see 2.1 Storage). Note that the FAIR principles should not be confused with the openness of a data set. Even data that are not publicly available (i.e. open) can be considered FAIR if this is warranted by e.g. privacy concerns.

A key element in producing FAIR data is proper data management. The YOUth data pose various data management challenges. For example, due to the wide range of measurements, the YOUth data are composed of a wide variety of data sources and formats. Some of these data, such as those obtained through questionnaires, can be stored in tabular data storage solutions (i.e. databases), while other data, such as videos of parent-child interactions and MRI scans, are not suited for tabular storage. In addition, biological samples are collected, which have to be stored physically. The management of the YOUth data is further complicated by the fact that the data collection effort spans across different legal entities. The research data are not just collected by YOUth researchers at our CRC, but also by partnering academic institutions, both regionally (e.g. MRI scans at the University Medical Center Utrecht (UMCU)) and nationally (e.g. a food questionnaire administered by Wageningen University (WUR)). This imposes additional regulatory burdens on the data collection, such as data processing agreements. Furthermore, the size, continuity and duration of the data collection demands high levels of storage space, throughput, reliability and durability from the data infrastructure. An estimated 63 GB of data is collected per week, with a projected total size of approximately 26 TB.

**Fig. 1:**
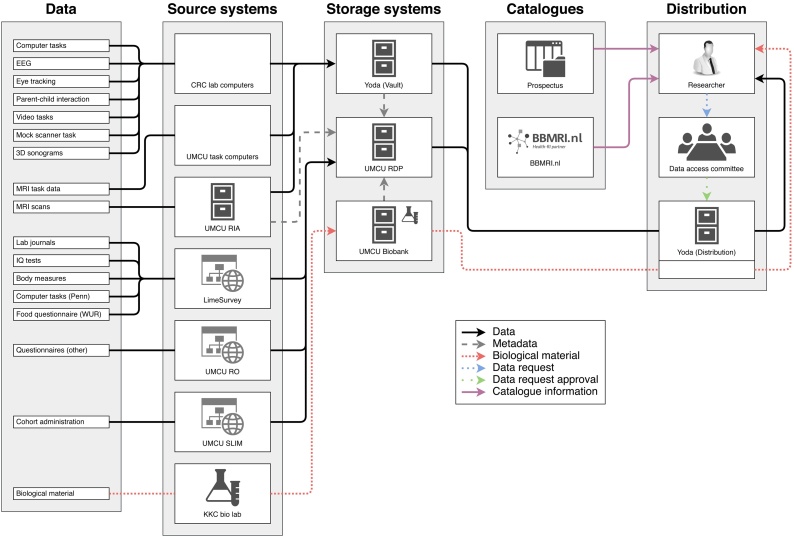
Overview describing the systems composing the infrastructure and the flow of data through these systems. Note that metadata of all the data are stored in the RDP and that the RDP sources these metadata from the repositories in which the data are held. The RIA→RDP metadata pipeline represents the use of a pre-existing metadata connection between these systems, capable of extracting additional useful metadata computed by the RIA. Abbreviations: CRC = Child Research Center; UMCU = University Medical Center Utrecht; SLIM = Study Logistics and Information Manager; RO = Research Online; RDP = Research Data Platform; WUR = Wageningen University; RIA = Research Imaging Architecture.

The topics to address in developing the data infrastructure itself concerned the *storage* of the data from source systems into centralized data repositories, the *structure* in which the source data are stored, the integrity of the *data transfer*, and the *integration* of the different storage systems into a unified whole (so as to achieve centralized overview and control of the data collection effort). An overview of our data infrastructure is found in Fig. 1. The topics addressed in further facilitating data FAIRness concerned to use of interoperable data formats, enabling meta data aggregation, providing a data access protocol, automating the handling of data requests, and facilitating data publication. All of these aspects will be covered in this section.

### 2.1 Storage

We sample tabular data (e.g. questionnaire responses), non-tabular data (like EEG or MRI measurements), and biological material, each requiring a dedicated storage solution. A facility for the storage of biological samples was already available in the Utrecht Biobank. Tabular data are stored in the UMCU Research Data Platform (RDP), which is part of the UMCU’s enterprise data warehouse solution, allowing us to extend pre-existing business intelligence capacities for advanced querying and selection of the data. For non-tabular data, ITS—informed by the needs of the YOUth study—developed Yoda,^1^ an open source^2^ research data management system based on iRods,^3^ that facilitates the research cycle from data storage, to research collaboration, to eventual publication of data sets with appropriate metadata (Smeele and Westerhof, 2018). Yoda compartmentalizes data into areas for data intake, collaboration (the research area) and permanent storage (the vault). The UMCU already operates a facility for the storage and processing of imaging data (the Research Imaging Architecture, UMCU RIA). This facility uses the open source imaging informatics platform XNAT (Marcus et al., 2007). Imaging data and metadata from this system are copied to Yoda and the RDP respectively, for permanent storage.

### 2.2 Structure

Data storage needs to be organized in a way that optimizes findability and retrieval. This is of fundamental importance, for without properly organized data, other requirements, such as data removal requests, cannot be met. In consultation with ITS, the YOUth data are organized by means of four characteristics: wave, experiment code, pseudocode (participant identification code) and version. The wave is the life-stage at which the participant visits us (e.g. at 5 months or 12 years of age), the experiment code is a shorthand for the experiment name (e.g. “infsgaze” for the Infant gaze cueing eye-tracking experiment), the pseudocode is a code consisting of a letter that identifies the subcohort (A for Child & Adolescent and B for Baby & Child) followed by a five-digit random number that uniquely identifies a participant, and the version indicates the version of a data set (e.g. raw data as obtained through the measurement computer or data further processed from these raw data). Since the participant can only take part in each experiment once per wave, the WEPV (wave, experiment, pseudocode, version) combination can uniquely identify a data set. The internal identifiers are thus assigned to (yet) unpublished datasets. When data sets are published, they are supplied with a globally unique identifier in the form of a DOI (of which the URL is pointing to a Yoda landing page). These DOIs are exposed through DataCite.

### 2.3 Transfer

As our data are sampled through different source systems (such as lab computers and online questionnaires) and need to be transported to their centralized data repositories for permanent storage, procedures are required that safeguard the integrity of the data transfer. We have developed such procedures, tailored to each data source. For example, for the non-tabular data collected through lab computers at the CRC, a script was written that calculates a cryptographic hash^4^ for each data file on the lab computer and then compares these hashes against a list of hashes of files stored in the Yoda vault. Files of which the hash does not match an entry on this list (i.e. that are not yet present in the vault) are then copied to the Yoda intake area. Files that do match are deleted from the lab computer. A YOUth data manager then reviews the copied files (e.g. checking whether all expected files are present, whether the file sizes are within the expected range, and whether they are properly named). This review has been largely automated through scripting. When anomalies do occur, they are corrected by a data manager in trivial cases (e.g. typos) or a researcher responsible for a particular subset of the measurements included in the cohort (the domain-responsible researcher, see Section 4) may perform a more in-depth review of the staged files. Once approved, the data are moved to the Yoda vault for permanent storage.

### 2.4 Integration

As explained above and shown in Fig. 1, the YOUth data are collected at various sources, labelled with a unique identifier (the WEPV code) and aggregated into three separate storage systems (Yoda, UMCU RDP and the Utrecht Biobank). The distributed nature of the data collection and storage is advantageous because it is optimally suited to the storage demands of each of the data types that we collect (non-tabular, tabular, biological). However, it lacks centralized overview and control of the entire cohort.

We solved this by adding two additional components. First, we used the SLIM (Study Logistics and Information Manager) cohort administration system, developed and operated by the UMCU. This system enables us to survey and control the data collection and to coordinate communication with our participants. Second, we aggregated the metadata of all collected data into the RDP. We thus created a centralized database with an overview of all data collected, which we can subsequently query to retrieve specific information about our data (e.g. how many MRI scans do we have of children whose mothers smoked during pregnancy).

### 2.5 Interoperability

The variety of measurements within YOUth come with a variety of data formats in which the data are stored. To ensure interoperability and long-term accessibility, we use non-proprietary output data formats preferred by the Data Archiving and Network Services organization (DANS) of the Royal Netherlands Academy of Arts and Sciences (DDI Alliance, 2018) and by the UK Data Service (UK Data Service, n.d.). Where this is not possible, the output data are stored in (open or closed source) proprietary data formats. If possible, data that are stored in proprietary data formats are converted to a non-proprietary data format after initial storage, and then stored alongside the original data. For example, in the case of 3D ultrasound data, the data are converted from 4DView to 3D-DICOM.

### 2.6 Metadata aggregation

YOUth data may be requested for use by researchers (see our Data Access Policy^5^). YOUth data can help answer many different kinds of research questions, but for researchers to be able to find out whether YOUth meets their needs, the data must be *findable* (by a computer) in the first place. Detailed, well-structured and machine-readable metadata are key to increasing the findability and the assessment of the reusability of data. Metadata with respect to research data consist of any information about the context in which those data were produced, e.g. organizational information about the cohort itself or the researchers involved. The metadata will also allow a deeper understanding of the data itself by providing relevant information about the experimental setup. This is, for example, realized by including (DOI-based) references to important underlying papers, i.e. articles that provide insight into the rationale behind the experiment that is actually performed. Furthermore, the metadata provide an unambiguous identification of equipment used, a description of the measurement methods that were applied, and keywords for the identification of phenomena involved in the experiment.

The size of the data and name of the measurement are examples of common metadata. High-quality metadata that go beyond common descriptors like these can open up a data set to novel use cases. Such metadata can, as explained in the former paragraph, provide information on the circumstances in which data were collected (e.g. the exact date, time and duration of task administration or the room temperature during administration), technical information (e.g. the make, model and key specifications of an MRI scanner) or detailed descriptive tags (e.g. “nicotine use” or “quality of sleep” indicating the information available in a particular questionnaire).

High-quality metadata are available for all experiments performed. However, they are currently mostly scattered through the Yoda file system in the form of folder names, settings files, and metadata headers for specific binary formats. Therefore, in collaboration with the metadata specialist of the Utrecht University Library, we developed a script to aggregate, validate and store our metadata in an explicit metadata structure, thereby allowing a mapping towards the Data Documentation Initiative (DDI, see also below) standard where applicable. This script runs on the data in the Yoda vault and does three things. First, it extracts metadata from various sources (e.g. WEPV information from the folder name and eye tracker device identifiers from settings files). Next, it validates the extracted metadata against a predefined schema (using JSON Schema^6^) which determines whether the values are valid. For example, the pseudocode (i.e. the P in WEPV) should start with a sub-cohort identifier (A or B) followed by a 5-digit string. Metadata values that do not pass validation are reported to the YOUth data manager for investigation and repair. Finally, if the data passes validation, they are written to the Yoda vault in the machine- and human-readable JSON data format in accordance with the predefined schema. This ongoing metadata aggregation process does not only improve the findability of our data, but it can also be useful in other ways. For example, it can help us to be better prepared for handling deficiencies in experimental software or equipment discovered after data have been collected. If for instance it were to be discovered that one of our EEG setups suffered a hardware defect, we could quickly find affected data and mark these for repair (if possible) or exclusion.

Until now, the improvement of our metadata has mainly increased the internal findability of our data, enabling our data managers to respond to a large variety of data requests. Although YOUth is indexed in domain-appropriate public catalogues that foster external findability (such as BBMRI^7^), more detailed metadata, as described above, are not yet externally findable. We plan to address this through the development of an interface by which external researchers can access these detailed metadata. This will save both the external researcher and the YOUth data manager time and effort, because the external researcher does not have to pass on a query to the data manager but can query the metadata him- or herself. External findability, reusability and interoperability may further be improved by adhering to standardized terminologies, i.e. controlled vocabularies. Controlled vocabularies solve the problem of the use of different terms to mean the same thing. For example, functional MRI data may be tagged as "fMRI" or as "MRI (functional)" according to the preference of the researcher responsible for that data. This lack of uniformity (either within a data set or between different studies) reduces findability. Different domain-specific metadata standards employ different vocabularies. An example of a widely used set of controlled vocabularies within the social sciences is the one published by the Data Documentation Initiative (DDI Alliance, 2018) that is currently in use by a number of European national social sciences data archives. At the time of writing we were notified that a grant was awarded^8^ to develop a digital infrastructure for harmonized metadata from six longitudinal youth cohorts in the Netherlands, including YOUth. This will allow us to take major steps in increasing the findability and interoperability of our data and the data from these other cohorts (all part of the Consortium on Individual Development, CID).

### 2.7 Data access protocol

The accessibility of the YOUth data is necessarily restricted. To clarify exactly under which circumstances, terms, and conditions access to (subsets of) our data can be granted, we have written and published a data access protocol. When (internal or external) researchers wish to request YOUth data, they fill out our data request form, which then goes through an extensive evaluation process by the YOUth Executive Board, the data manager, and the Data Management Committee. Data requests are assessed on whether they fall within the YOUth framework (i.e. relate to brain and/or behavioural development, preferably to social competence and/or self-regulation), and within the limits of the informed consent provided by the participants of YOUth. Moreover, requests are evaluated in terms of the specificity of the research question, hypothesis, proposed method, and analysis plans, and on whether the requested (combination of) data may pose risks to the privacy of participants. Access to biological materials can only be granted after additional approval from the Biobank Review Board (Toetsingscommissie Biobanken) of the UMC Utrecht. A detailed description of (further) terms, conditions and procedures is available in the YOUth Data Access Protocol.^9^

### 2.8 Automating the handling of data requests

As the scope of the YOUth cohort is broad and its data is thus of potential interest to a broad range of researchers, we need to be prepared for frequent data requests. In addition, as outlined above and detailed in the YOUth Data Access Protocol, the handling of a data request is a multi-step procedure involving multiple actors.

To streamline the handling of data requests and increase the ease of accessibility of the YOUth data, we developed an automated data request processing system as an extension to Yoda (expected to be available summer 2020). This system implements the flow of the procedures specified in the YOUth Data Access Protocol. It allows researchers to submit data requests online, and facilitates the actions requested from the people involved in the evaluation and transfer procedures. By developing this system as an extension to our main data storage facility, we are able to combine the request, staging and transfer of data within a single system, simplifying the process for all actors involved. The requesting researcher is automatically kept up to date on the progress of the data request. All information and documents needed to establish an audit trail are captured by the system.

### 2.9 FAIR principles implementation overview

**Table:** 

Principle	Implementation
F1. (Meta)data are assigned a globally unique and persistent identifier	Published data sets are assigned a DOI.
F2. Data are described with rich metadata (defined by R1 below)	Currently the assigned metadata is DataCite 4.x compliant. However, as part of the grant referred to in Section 2.6, a transition to DDI LifeCycle 3 has been started which will result in a large findability increase through the extensive use of standard community-driven controlled vocabularies.
F3. Metadata clearly and explicitly include the identifier of the data they describe	Metadata entries in the UMCU RDP include the WEPV code. The metadata of published data sets list the DOI of the data.
F4. (Meta)data are registered or indexed in a searchable resource	WEPV metadata are stored in the UMCU RDP, which is a searchable resource. Additional metadata are stored in JSON format in Yoda (see Section 2.6). Metadata of published packages will be harvestable (machine-readable) via OAI-PMHa endpoints and are exposed in the DataCite format.
A1. (Meta)data are retrievable by their identifier using a standardized communications protocol	Data sets are retrievable from Yoda through a WebDAV and a HTTP interface, though only in the case of published data is it retrievable using a DOI. See also F4.
A1.1 The protocol is open, free, and universally implementable	WebDAV and HTTP are open, free, and universally implementable. JSON is currently used internally. We are investigating future use of JSON-LD or RDF. The OAI-PMH endpoints will expose XML-data. The data access protocol is publicly available.
A1.2 The protocol allows for an authentication and authorization procedure, where necessary	This is necessary. The authorization procedure is described in the publicly available data access protocol. Authentication is handled by Yoda through use of the open WebDAV protocol (and therefore also available to machines).
A2. Metadata are accessible, even when the data are no longer available	For published data sets, a landing page with metadata about the data set remains available when the data set is no longer available. Metadata within the RDP remains available after data removal (e.g. after consent withdrawal).
I1. (Meta)data use a formal, accessible, shared, and broadly applicable language for knowledge representation.	As a result of the transition to DDI LifeCycle 3 (see F2), the semantic representation of our data will come to be based on a broadly applicable common language (e.g. through the use of harmonized variables and terminologies).
I2. (Meta)data use vocabularies that follow FAIR principles	Controlled vocabularies and reference improvements are part of the CID metadata harmonization plans. See Section 2.6.
I3. (Meta)data include qualified references to other (meta)data	
R1. Meta(data) are richly described with a plurality of accurate and relevant attributes	Yes. See Section 2.6 for examples.
R1.1. (Meta)data are released with a clear and accessible data usage license	Published data sets on Yoda have a mandatory License field.
R1.2. (Meta)data are associated with detailed provenance	Detailed provenance information (e.g. laboratory setups or test administration protocols) is available for all data.
R1.3. (Meta)data meet domain-relevant community standards	The initiated transition from the domain-agnostic DataCite descriptions to DDI LifeCycle 3 (see F2) incorporates subdiscipline-specific methodology descriptions and introduces a wider use of community-driven vocabularies. Moreover, the more detailed information about e.g. constraints and variables used has a large positive impact on the reusability as a whole.

Open Archives Initiative Protocol for Metadata Harvesting. See https://www.openarchives.org/pmh/.

## 3 Safe data

The safety of our participants and their data are our highest priority, especially since we are collecting data from children. In this section, we describe how we protect their privacy, confidentiality, and safety throughout and after their participation in YOUth.

### 3.1 Data protection

The data from the YOUth study cannot be stored anonymously since the study involves subjects repeatedly visiting the research centre. Therefore, a participant administration has to be kept for purely logistical reasons. A first safeguard of the confidentiality of the data we collect is that the systems developed and operated by ITS (i.e. Yoda) conform to the security requirements specified by the Utrecht University Information Security Policy (Utrecht University, 2015), which in turn is based on the ISO/IEC 27001 IT security standard (International Organization for Standardization, 2013). Yoda is subject to periodic professional internal and external security audits. These marks of mature information security practices provide us and our participants with reasonable assurance of the confidentiality of the YOUth data. The systems operated by the UMCU are likewise compliant with current laws and regulations concerning data protection. More specifically, given that the storage of participant identities could not be avoided, we sought to implement this requirement as safely as possible while maintaining an efficient data collection workflow. Guided by the privacy-by-design approach to systems engineering (Cavoukian, 2011), we separated the cohort administration system (SLIM) from the data collection systems. While SLIM uses a participant number to identify a participant, a different, randomly generated persistent identifier (which we call a "pseudocode") is used to identify the participant during data collection. The participant number and the pseudocode cannot be derived from one another. The only means of linking participant information to collected data is through the use of a linking table (stored in SLIM), access to which is limited to the YOUth data managers and a select few other actors. Thus, information that directly identifies participants is never stored together with research data, providing a barrier against the accidental release of personal data, while at the same time allowing for participants to be linked to research data if necessary.

Besides these technical data safety measures, data transfer agreements are used to restrict the use of data by third parties, legally binding them to only process the data on secure systems under their control and to securely destroy the data when they have served their purpose (e.g. the production of research output).

### 3.2 Participant safety

All our procedures are approved by the institutional review board of the UMCU. As the YOUth cohort involves many different tasks and measurements, we split up the approval into a framework protocol and an amendment to the framework protocol for each of the waves. This construction allows us to modify our protocols in order to keep up with changing privacy legislation and to make necessary adjustments to our tasks and measurements over the course of many years of data collection. In addition, we developed a series of Standard Operating Procedures (SOP).^10^ These SOPs instruct YOUth staff step by step on how to act in specific situations and are developed in line with current medical, ethical and legal regulations. SOPs are available for, among others, adverse events, good clinical practice, data breaches and informed consent.

### 3.3 Handling consent revocation and data removal requests

The GDPR legally requires us, under certain conditions, to comply with data removal requests (GDPR art. 17). Participants are also free to revoke their consent and to require removal of their data. Consent revocation forms are available to participants (on paper and on the YOUth website), allowing them to revoke their consent temporarily or permanently, and allowing them to decide whether the data already collected are allowed to remain available for study or should be destroyed. However, in order to make verification of published results possible, data that have been used for published research before the removal request arrives will not be destroyed but marked as unavailable for future research instead. A withdrawal can be permanent (for all future waves) or temporary (for a specific wave), and can apply to one or more family members. The consent revocation is administered in SLIM and in the RDP, the latter enabling the data manager to remove data from source systems when requested.

### 3.4 Minimizing data exchange

The post-GDPR world has renewed attention for and concerns about data privacy. Simultaneously, the digital threat landscape has gotten more hostile as opportunities to maliciously exploit intrusions of internet-connected systems have evolved (Accenture, 2019). Furthermore, complex geopolitical situations can complicate or even prohibit certain international academic cooperations (van Deursen and Kummeling, 2019). These considerations increase the tension between the open science ideal and the necessity and practicalities of keeping privacy-sensitive data confidential. To increase our data safety even further, we are therefore also actively investigating technical solutions that enable analyses of the YOUth data by third parties without the necessity of handing them copies of the data. Two solutions in particular have our attention: virtual research environments (VREs) and the personal health train approach (Dutch Techcentre for Life Science, n.d.). VREs are, broadly speaking, data collaboration environments under an organization’s own control equipped with analysis software, made accessible to trusted third-party researchers. While VREs inherently cannot prevent data exfiltration, they greatly reduce the risk of accidental data compromise by third-party researchers.

The personal health train approach goes a step beyond VREs in terms of data security by preventing the third party from directly interacting with the data at all. Instead, the desired analysis is performed where the data reside (often distributed across multiple storage locations, hence the image of a “data train” travelling along these locations), after which the results of the analysis are transmitted to the researcher. As an addition to the PHT approach, we propose that a data set may be made publicly available that is identical to the real data set in structure and file formats (and, if possible, in statistical properties), but whose actual data consists of (plausible) randomly generated data or data from a set of test runs. This allows third parties that are interested in testing particular hypotheses against the YOUth data to develop appropriate analysis scripts in the absence of the actual data. The hypothesis and accompanying analysis script are then submitted to YOUth for review and, if approved, are granted execution privilege, after which the analysis results are returned to the third party.

## 4 High-quality data

Ensuring that our data are of high quality requires the measurements and experiments to not only be well-constructed, but also their data output to be subject to regular data quality control. Three aspects of the YOUth cohort make this especially important. First, being a longitudinal study, we are particularly interested in differences between waves. Therefore, any change to a task or experimental setup may confound the interpretation of differences found between waves. Second, due to the complexity of some of the experimental setups and equipment, there are risks of unintentional adjustments or defects during data collection. These can degrade or compromise the collected data. Third, the rapid and continual pace of inclusion means that defects of experimental setups have near-immediate consequences.

The YOUth cohort includes experiments and measurements from across the spectrum of cognitive (neuro)science. While responsibility for the logistic quality controls of all data (the number of files, file sizes, etc.) is centralized in the data manager, it is undesirable to delegate all quality controls to a single person or group because of the domain-specific expertise required. Therefore, we designate domain-responsible researchers to monitor the quality of output data falling within their domain (e.g. eye-tracking, EEG or MRI). The implementation of this process is designed by the domain-responsible researchers themselves, using their expertise and the common practices in their respective fields.

Here we briefly describe the steps that have been taken for the eye-tracking domain by the domain-responsible researcher, as an example of how our protocols are implemented and revised to ensure high-quality data. Obtaining high-quality data is a multi-step process, which includes at least (1) the optimization of the experimental setups, (2) the training of the research assistants, (3) the monitoring of the quality of the obtained data, and (4) the implementation and registration of adjustments to experimental setups or data-collection procedures. Specific steps that have been implemented for the eye-tracking domain are:

• The development and implementation of a dedicated eye-tracking setup capable of obtaining high-quality data with multiple age groups. The setup is explained in detail in Hessels and Hooge (2019, this issue)
• The training of research assistants as to the proper use of the experimental setup. This includes watching a series of instruction videos and observing measurements conducted by more experienced research assistants.
• The creation of an open atmosphere in which research assistants are comfortable sharing potential anomalies or errors to the YOUth manager of logistics, who will then discuss these with the domain-responsible researcher.
• Online monitoring during the recording of the estimated gaze position on the computer screen, using a separate screen connected to the stimulus computer. This allows research assistants to estimate the data quality as they are being recorded.
• In-person observation of research assistants' measurements by the domain-responsible researcher or a delegated party (about once every two months).
• Control of the newly collected data using eye-tracking specific indicators of data quality (see Hessels and Hooge, 2019, this issue), both periodic (once every couple of months) and after changes in the experimental setup, among which (but not limited to) changes in the measurement procedure, software, lab location, lighting, etc.

For all domains, periodic quality control reports are written by the domain-responsible researchers, documenting any potential anomalies and proposing potential countermeasures. These reports are delivered to the YOUth executive director so that centralized overview and control of the data quality is maintained.

Although quality control is executed rigorously in this manner, fully automated quality control is one of our ambitions. Automated data quality control integrated into the entire data pipeline (e.g. through scripts placed on a dedicated system that computes data quality metrics for all new data recorded) would circumvent the manual step of having to access new data by the domain-responsible researcher to run quality control scripts, and would allow researchers and research assistants to continuously monitor data quality. In some of our domains, great steps have already been taken to implement this. For example, a data-quality control pipeline as just described is already operational within the MRI domain, including interactive reports of quantitative and qualitative measures generated immediately after data acquisition (an extensive description of which can be found in Buimer et al., 2020, this issue). The experience with implementing automated quality control gained in these domains will help us establish this in our other domains, leading to a gradual widespread implementation.

## 5 Discussion

In this paper, we described how YOUth, as a large-scale longitudinal cohort study collecting vast amounts of sensitive data, aims to produce and safely store FAIR, high-quality research data. We described the technical aspects of our data and of setting up procedures and a data infrastructure that support these aims. Here, we will reflect on the organizational aspects that are conducive to the success of setting up such an enterprise, and we consider the financial challenges posed by individual studies investing in sustainable science.

Our research project is embedded in different organizations. A crucial prerequisite to our success was to have partner organizations with a clear ambition to support proper data management and accessibility, and the means and institutional support to realize this ambition. Important aspects of such institutional support are a dedicated research IT division and high-quality data managers^11^ that pro-actively work to improve all aspects of the data management. Data managers will, through daily interaction with the data and the infrastructure, be well-positioned to spot potential improvements to the FAIRness, quality and safety of the data and data infrastructure. A data manager is also in close contact with researchers and therefore able to act as a bridge between the scientific domain and that of IT. Also essential is frequent and extensive contact and cooperation between the organizational units involved. This is especially important when previous collaboration between such organizations has been limited or short-lived, as in these cases a project cannot fall back on established routines and will have to pioneer many aspects of the collaboration. Identifying key players within partner organizations at both the executive and the management level and establishing good relationships with them is essential to efficient cooperation. Throughout these contacts, an attitude of patience, persistence, and forgiveness helps to overcome organizational differences and misunderstandings.

In addition to these human aspects, there are financial aspects to consider. While practicing open and sustainable science is gaining ground in academia, the substantial financial investments that are required for large cohort studies to do so are lacking. Such studies are usually dealing with sensitive personal data that cannot be made publicly available and therefore need to invest in long term management of the data infrastructure and procedures regulating the sharing of data. More (long term) funding for projects that produce large amounts of high-quality, FAIR data that is securely stored and managed is therefore crucial if we want to make science more sustainable. A return on such investments could at least partially be made by reuse of the data. For many academics in human research the collection of new data is a standard element of the scientific cycle. While for many research questions new studies may indeed be necessary, there are also many research questions for which the reuse of large and high-quality (existing) datasets will be essential, for example in stimulating (multidisciplinary) cooperation. As a bonus, reusing data might cut the cost for data collection. Subsidising the implementation of safe, FAIR and high-quality datasets is therefore in the end a scientifically, and potentially also economically, sound investment.

## Funding

The YOUth cohort is part of the Consortium on Individual Development (CID), which is funded through the Gravitation programme of the Dutch Ministry of Education, Culture, and Science and the Netherlands Organization for Scientific Research (NWO grant number 024.001.003).

## Declaration of Competing Interest

The authors declare that they have no known competing financial interests or personal relationships that could have appeared to influence the work reported in this paper.

## Footnotes

1 Originally an acronym for YOuth DAta. Now an acronym for YOur DAta, because deployment has expanded beyond YOUth.

2 Available at https://github.com/UtrechtUniversity/yoda-uu-nl/.

3 iRODS is an open source data management solution. See https://irods.org/.

4 A hash function takes data and returns a short value (often a string of characters) that is unique to the data given. If the data changes (even a single bit), the return value changes. Thus, it can be used to identify a piece of data. We used the SHA-256 hash function (see https://en.wikipedia.org/wiki/SHA-2).

5 https://www.uu.nl/en/research/youth-cohort-study/data-access.

6 See https://json-schema.org/.

7 https://catalogue.bbmri.nl/menu/main/app-molgenis-app-biobank-explorer/collection/bbmri-eric:ID:NL_CBB:collection:aaaac2mrmv2ygacqk2mpm4yaae.

8 https://individualdevelopment.nl/2020/06/11/funding-for-harmonized-metadata-infrastructure/.

9 Available at https://www.uu.nl/sites/default/files/dataaccessprotocol_youth_191029.pdf.

10 Available in Dutch at https://www.uu.nl/en/research/youth-cohort-study/youth-standard-operating-procedures.

11 Estimating the number of full-time data managers required for a given research project is non-trivial and outside of the scope of this article. As a rough indication, we have estimated 1.6 FTE for all data manager tasks and responsibilities (foreseen and unforeseen) and have been able to remain operational at 1.2 FTE.

## References

[bib0005] Accenture, 2019. The Cost of Cybercrime: Ninth Annual Cost of Cybercrime Study. https://www.accenture.com/_acnmedia/pdf-96/accenture-2019-cost-of-cybercrime-study-final.pdf (accessed 28 February 2020).

[bib0010] Buimer Elizabeth E.L., Pas Pascal, Brouwer Rachel M., Froeling Martijn, Hoogduin Hans, Leemans Alexander, Luijten Peter, van Nierop Bastiaan J., Raemaekers Mathijs, Schnack Hugo G., Teeuw Jalmar, Vink Matthijs, Visser Fredy, Hulshoff Poll Hilleke E., Mandl René C.W. (2020). The YOUth cohort study: MRI protocol and test-retest reliability in adults. Dev. Cogn. Neurosci..

[bib0015] Cavoukian Ann (2011). Privacy by Design in Law, Policy and Practice: A White Paper for Regulators, Decision-makers and Policy-makers.

[bib0020] DDI Alliance, 2018. Controlled Vocabularies - Overview Table of Latest Versions. https://ddialliance.org/controlled-vocabularies (accessed 28 February 2020).

[bib0025] Dutch Techcentre for Life Sciences, n.d. Personal Health Train. https://www.dtls.nl/fair-data/personal-health-train/ (accessed 28 February 2020).

[bib0030] Hessels Roy S., Hooge Ignace T.C. (2019). Eye tracking in developmental cognitive neuroscience – the good, the bad and the ugly. Dev. Cogn. Neurosci..

[bib0035] International Organization for Standardization (2013). Information Technology — Security Techniques — Code of Practice for Information Security Controls (ISO Standard no. 27001:2013).

[bib0040] Marcus Daniel S., Olsen Timothy R., Ramaratnam Mohana, Buckner Randy L. (2007). The extensible neuroimaging archive toolkit. Neuroinformatics.

[bib0045] Mons Barend (2018). Data Stewardship for Open Science: Implementing the FAIR Principles.

[bib0050] NWO, n.d. Open (FAIR) data. https://www.nwo.nl/en/policies/open+science/data+management/ (accessed 28 February 2020).

[bib0055] Onland-Moret N. Charlotte, Buizer-Voskamp Jacobine E., Albers Marieke E.W.A., Brouwer Rachel M., Buimer Elizabeth E.L., Hessels Roy S., de Heus Roel, Huijding Jorg, Junge Caroline M.M., Mandl René C.W., Pas Pascal, Vink Matthijs, van der Wal J.J.M., Hulshoff Poll Hilleke E., Kemner Chantal (2020). The YOUth study: rationale, design, and study procedures. Dev. Cogn. Neurosci..

[bib0060] Smeele Ton, Westerhof Lazlo R. (2018). Using iRODS to manage, share and publish research data: Yoda. iRODS User Group Meeting 2018 Proceedings.

[bib0065] UK Data Service, n.d. Recommended formats. https://www.ukdataservice.ac.uk/manage-data/format/recommended-formats (accessed 28 February 2020).

[bib0070] Utrecht University (2015). Informatiebeveiliging binnen Universiteit Utrecht: Deel 1.

[bib0075] Utrecht University Open Science Task Force, 2018. Utrecht University Open Science Programme 2018–2021. https://www.uu.nl/sites/default/files/utrecht-university-open-science-programme.pdf (accessed 28 February 2020).

[bib0080] van Deursen Stijn, Kummeling Henk (2019). The New Silk Road: a bumpy ride for Sino-European collaborative research under the GDPR?. High. Educ..

[bib0085] Wilkinson Mark D., Dumontier Michel, Aalbersberg Ijsbrand J., Appleton Gabrielle, Axton Myles, Baak Arie, Blomberg Niklas, Boiten Jan-Willem, da Silva Santos Luiz B., Bourne Philip E., Bouwman Jildau, Brookes Anthony J., Clark Tim, Crosas Mercè, Dillo Ingrid, Dumon Olivier, Edmunds Scott, Evelo Chris T., Finkers Richard (2016). The FAIR Guiding Principles for scientific data management and stewardship. Sci. Data.

